# Alcohol Consumption Patterns during COVID-19 Lockdown and Their Relationship with Perceived Immune Fitness and Reported COVID-19 Symptoms

**DOI:** 10.3390/healthcare9081039

**Published:** 2021-08-13

**Authors:** Agnese Merlo, Pauline A. Hendriksen, Noortje R. Severeijns, Johan Garssen, Gillian Bruce, Joris C. Verster

**Affiliations:** 1 Division of Pharmacology, Utrecht Institute for Pharmaceutical Sciences, Utrecht University, 3584CG Utrecht, The Netherlands; a.merlo@uu.nl (A.M.); p.a.hendriksen@students.uu.nl (P.A.H.); n.r.severeijns@students.uu.nl (N.R.S.); j.garssen@uu.nl (J.G.); 2 Global Centre of Excellence Immunology, Nutricia Danone Research, 3584CT Utrecht, The Netherlands; 3 Division of Psychology and Social Work, School of Education and Social Sciences, University of the West of Scotland, Paisley PA1 2BE, UK; gillian.bruce@uws.ac.uk; 4 Centre for Human Psychopharmacology, Swinburne University, Melbourne, VIC 3122, Australia

**Keywords:** alcohol, mood, stress, perceived immune fitness, COVID-19

## Abstract

Since the outbreak of the 2019 coronavirus (COVID-19) pandemic, lockdown periods have been installed to counteract the spread of the virus. These lockdowns, characterized by social isolation, have been associated with mood changes and increased stress. Individuals have used various strategies to cope with the negative effects of being in lockdown, including increasing the frequency and quantity of alcohol consumption. The aim of this study was to investigate mood before and during lockdown of individuals who reported consuming more, less, or the same amount of alcohol during lockdown, and examine how this impacts and perceived immune fitness and the presence and severity of COVID-19 symptoms. Analysis included a sub-sample from the ‘Corona Lockdown: how fit are you?’ (CLOFIT) study, comprising N = 761 participants who reported consuming alcohol in 2020. The results of the online survey showed that half of the participants did not alter their weekly alcohol consumption during lockdown (50.4%), whereas 25.9% of drinkers reported a reduction and 23.8% reported an increase in weekly alcohol consumption. Compared to individuals that did not alter their drinking behaviour, both increased and reduced alcohol consumption during lockdown was associated with poorer mood and higher stress levels. Increased alcohol consumption was associated with significantly reduced perceived immune fitness and a high presence and severity of COVID-19 symptoms. This effect was not significant among individuals with reduced or unaltered alcohol consumption.

## 1. Introduction

The outbreak of the coronavirus disease 2019 (COVID-19), subsequently declared a global pandemic by the World health Organisation (WHO), led to several social and economic implications, impacting individuals’ behavioural patterns and psychological wellbeing [1,2]. Studies investigating the psychological impact of lockdown or quarantine as during COVID-19 found significant associations with psychosocial distress, including anxiety, depression, stress, and a decrease in mood [3,4,5]. A related public health concern that emerged during this pandemic is the reporting of increased alcohol consumption. Indeed, previous research suggests that lockdowns and stay at home mandates negatively affect psychological wellbeing and may lead to increased substance misuse, including alcohol consumption [6,7]. Increased alcohol consumption may have a direct negative effect on the susceptibility for COVID-19. For example, a systematic review and meta-analysis by Simou et al. [8] evaluated 13 studies and concluded that higher levels of alcohol consumption were significantly associated with an increased risk of acute respiratory distress syndrome (ARDS). Increased alcohol consumption has been shown to have negative effects on the immune fitness, i.e., the capacity of the body to respond to health challenges such as viral infections, thus making individuals more susceptible to viral exposure and infections [9,10], as well as resulting in an increased need for ventilation and prolonged stay in intensive care units [11,12]. Thus, it is important to understand the impact of isolation and quarantine on drinking habits, as frequency and quantity of alcohol use can increase and in turn compromise the immune system response and thereby susceptibility to viral infection.

In several countries, studies have been conducted to investigate the impact of COVID-19 lockdown on alcohol consumption. A review of the literature reveals that the outcomes of these studies are inconclusive. That is, for the sample as a whole, some studies report an increase in alcohol consumption [13,14,15,16,17], whereas other studies report a decrease in alcohol consumption [18,19,20], or no change [21]. For each of these findings, the authors hypothesize plausible causes. For example, an increase in alcohol consumption is often explained as a way of coping with lockdown-related stress and mood changes [14,22,23,24,25], or financial concerns, quarantine, or conducting work or studies from home [26,27,28]. A decrease in alcohol consumption is associated with the closure of pubs and restaurants [20,24,29]. However, with regard to the latter, it should be noted that despite the closure of pubs and restaurants during the pandemic, a rise in alcohol sales have been reported in various countries, including the USA [30] and the UK [31]. Others may have reduced their alcohol intake because they have a higher susceptibility for viral infection (e.g., medical risk groups such as individuals with diabetes), or adopted to a healthy lifestyle in order to reduce their chance of acquiring COVID-19 [22,26,32]. Alternatively, some studies identified subgroups within their sample that either consume more, less, or the same amount of alcohol during lockdown [13,22,24,25,26,30,33]. These results are likely more feasible, as not all individuals are similarly impacted by being in lockdown, and motives for alcohol consumption and drinking behaviours differ greatly among individuals [15,26,34,35].

In the Netherlands, a lockdown was enforced from the 15 March until the 11 May 2020. In our first analysis of the alcohol consumption data of the Dutch ‘Corona Lockdown: how fit are you?’ (CLOFIT) study we found no overall effect on alcohol consumption when comparing lockdown versus pre-lockdown weekly alcohol consumption [36,37]. However, in this study, the sample analysed for alcohol consumption was not a homogenous group, and included both participants that increased or decreased alcohol consumption. Merlo et al. [37] described a model linking increased alcohol intake with reduced perceived immune fitness and the increased presence and severity of COVID-19-related symptoms. It was shown that a decrease in mood and increased stress during lockdown had a negative impact on perceived immune fitness. In turn, reduced immune fitness, i.e., the capacity of the body to respond to health challenges such as viral infections, makes individuals more susceptible to viral exposure and infections, such as COVID-19.

The aim of the current analysis was to further investigate alcohol consumption during lockdown, and the relationship with mood, stress, and perceived immune fitness, as well as their relationship with the presence and severity of COVID-19-related symptoms. It was hypothesized that both increased and decreased alcohol consumption are associated with poorer mood and increased stress as a result of the COVID-19 lockdown. Although effects on alcohol intake are the opposite for the groups that either increased or decreased their alcohol consumption, both act as a mechanism to cope with stress. It is further hypothesized that increased alcohol consumption is associated with poorer perceived immune fitness compared to the group of drinkers who reduced their alcohol use. In contrast, for the groups with reduced and unchanged alcohol consumption, no negative effect on immune fitness was expected. Lastly, it was hypothesized that the group who increased their alcohol consumption would report more and more severe symptoms of COVID-19 compared to the other groups.

## 2. Materials and Methods

Data of alcohol consumers who completed the CLOFIT study were analysed [36]. The CLOFIT study was an online survey which was conducted between 24 June and 26 July 2020. The aim of the survey was to examine the psychosocial and health consequences of the COVID-19 pandemic in the Netherlands. The survey included questions and scales for the period before the national lockdown (15 January–14 March 2020) as well as for the lockdown period itself (15 March–11 May 2020).

The survey was developed in SurveyMonkey, and participants’ recruitment was conducted via a Facebook advertisement. The study aimed at including the general Dutch population, and its advertisement targeted Dutch adults aged 18 years and older. Electronic informed consent was obtained from all participants, and the Ethics Committee of the Faculty of Social and Behavioral Sciences of Utrecht University granted ethical approval (approval code FETC17-061).

Demographics included age, sex, weight, and height. Body mass index (BMI) was computed. Participants reported the number of alcoholic drinks they consumed on average per week, and the number of days they consumed alcohol per week. Guidance was provided regarding drinking sizes and how to convert these into standardized units of alcohol (10 g). Mood was assessed via 1-item scales and included ‘anxiety’, ‘stress’ ‘depression’, ‘fatigue’, ‘hostility’, ‘loneliness’, and ‘happiness’. All items were scored on a scale ranging from 0 (absent) to 10 (extreme). Coping with stress was assessed with the corresponding subscale of the Fantastic Lifestyle Checklist [38,39]. Scores range from 0 to 8, with higher scores implicating greater ability of coping with stress. Perceived immune fitness during lockdown was assessed using a 1-item scale ranging from 0 (poor) to 10 (excellent), with higher scores indicating better perceived immune fitness [40,41]. The COVID-19 Symptoms Scale comprised nine items, including sneezing, running nose, sore throat, cough, and malaise/feeling sick, high temperature (up to 38 Celsius), fever (38 Celsius and higher), shortness of breath, and chest pain. The severity of each item could be rated as none (0), mild (1), moderate (2), or severe (3). The sum of the item scores is the COVID-19 Symptom Severity Score, with a possible range from 0 (no complaints) to 27 (severe complaints). In addition, the presence of COVID-19 symptoms was calculated by counting the number of symptoms with a score > 0.

Statistical analyses were conducted with SPSS (IBM Corp. Released 2013. IBM SPSS Statistics for Windows, Version 27.0. Armonk, NY, USA: IBM Corp.). Subjects were grouped according to their change in drinking behaviour during the lockdown period relative to the period before lockdown. The three groups consumed (1) less, (2) the same, or (3) more alcohol during the lockdown period. Mean and standard deviation (SD) were computed for all variables. Outcome measures of the 3 groups were compared using the independent samples Kruskal–Wallis Test, and Bonferroni’s correction was applied. Outcomes before and during lockdown were compared using the related samples Wilcoxon signed rank test. Differences were considered statistically significant if *p* < 0.05 (two-tailed).

## 3. Results

N = 761 participants completed the survey, with an age range of 18 to 94 years old (61.6% women). Participant demographics are summarized in Table 1.

**Figure 1:**
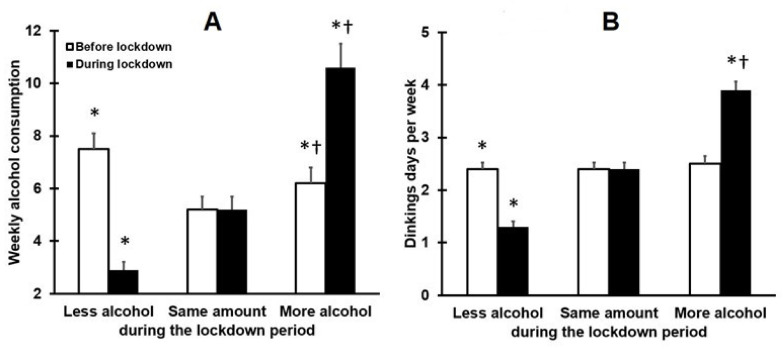
Weekly alcohol consumption and number of drinking days for subjects drinking more, less, or the same amount of alcohol during the lockdown period. Mean and standard error of weekly number of alcoholic drinks (**A**) and number of weekly drinking days (**B**) are shown. Nonparametric comparisons were conducted with the independent samples Kruskal–Wallis test. Paired comparisons (two-tailed) were adjusted for multiple comparisons by applying a Bonferroni’s correction. Differences were considered statistically significant if the adjusted *p*-value was < 0.05. Significant differences between less or more alcohol groups and the same amount of alcohol group are indicated by *. Significant differences between the less alcohol group and more alcohol group are indicated by †.

Participants were categorized according to their change in drinking behaviour during the lockdown period. It appeared that about half of the participants did not alter their weekly alcohol consumption during lockdown (50.4%), whereas 25.9% of drinkers reported a reduction in weekly alcohol consumption during lockdown, and 23.8% reported an increase in weekly alcohol consumption. The drinking behaviour of the three groups is summarized in Figure 1.

For the group that increased their alcohol intake during lockdown, the mean (SD) weekly alcohol intake increased from 6.2 (7.9) to 10.6 (11.9) units (*p* < 0.001). In the group that reduced their alcohol intake during lockdown, the mean (SD) weekly alcohol intake decreased from 7.5 (8.2) to 2.9 (4.0) units (*p* < 0.001). Relative to the pre-lockdown period, the percentage of at-risk drinkers (women > 7 drinks and men > 14 alcoholic drinks per week) approximately doubled from 14.2% to 31.0% in women and from 21.4% to 39.3% in men. Thus, around one-third of the overall sample exhibited at risk alcohol consumption levels during the lockdown period.

### 3.1. Drinking Outcomes and Mood

**Figure 2:**
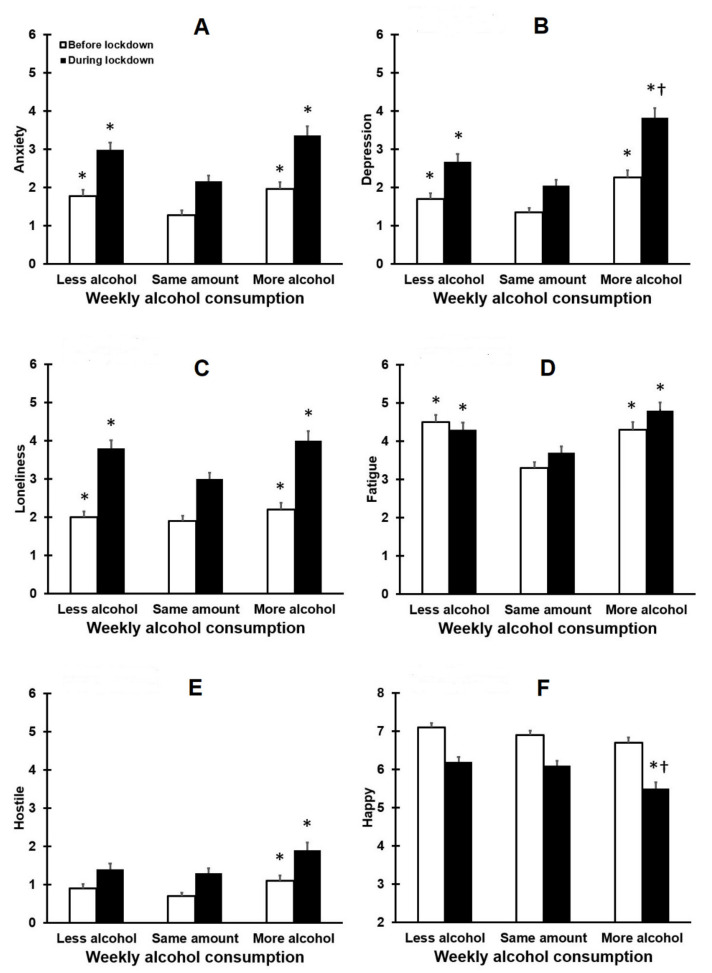
Mood before and during lockdown. Means and standard deviations are shown for anxiety (**A**), depression (**B**), loneliness (**C**), fatigue (**D**), hostile (**E**), and happy (**F**). Paired comparisons (two-tailed) were adjusted for multiple comparisons by applying a Bonferroni’s correction. Significant differences between less or more alcohol groups and the same amount of alcohol group are indicated by *. Significant differences between the less alcohol group and more alcohol group are indicated by †.

Mood changes were evaluated for drinkers consuming less, the same amount, or more alcohol during lockdown. Figure 2 summarizes the results.

Figure 2 shows that across the three groups, mood was significantly poorer during the lockdown period. Interestingly, compared to participants that did not alter their drinking behaviour, poorer mood was reported by both groups that consumed less or more alcohol consumption during lockdown. These mood effects were strongest in those who increased their alcohol consumption. The difference between the groups who consumed more or less alcohol reached statistical significance only for depression (*p* = 0.008) and happiness (*p* = 0.007).

### 3.2. Stress and Coping

**Figure 3:**
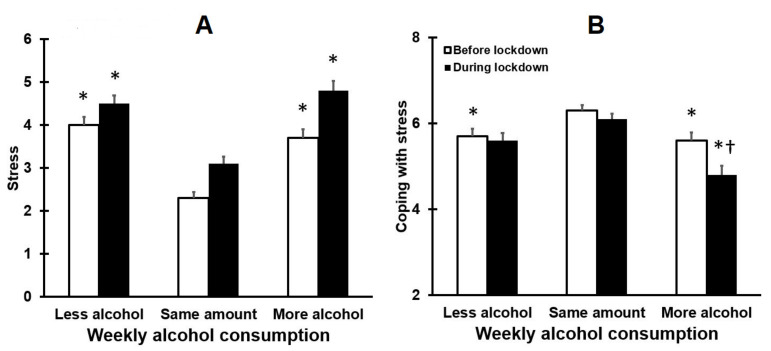
Stress and coping with stress before and during lockdown. Means and standard errors are shown for stress (**A**) and coping with stress (**B**). Nonparametric comparisons were conducted with the independent samples Kruskal–Wallis test. Paired comparisons (two-tailed) were adjusted for multiple comparisons by applying a Bonferroni’s correction. Differences were considered statistically significant if the adjusted *p*-value was < 0.05. Significant differences between less or more alcohol groups and the same amount of alcohol group are indicated by *. Significant differences between the less alcohol group and more alcohol group are indicated by †.

In line with the observations for mood, stress levels were significantly higher during lockdown. Compared to drinkers that did not alter their weekly alcohol consumption, stress levels before and during lockdown were significantly higher in the groups who consumed either less or more alcohol during lockdown (see Figure 3A). The group that consumed more alcohol during lockdown reported significantly more stress and was significantly less capable of coping with stress.

### 3.3. Alcohol Consumption and Perceived Immune Fitness

**Figure 4:**
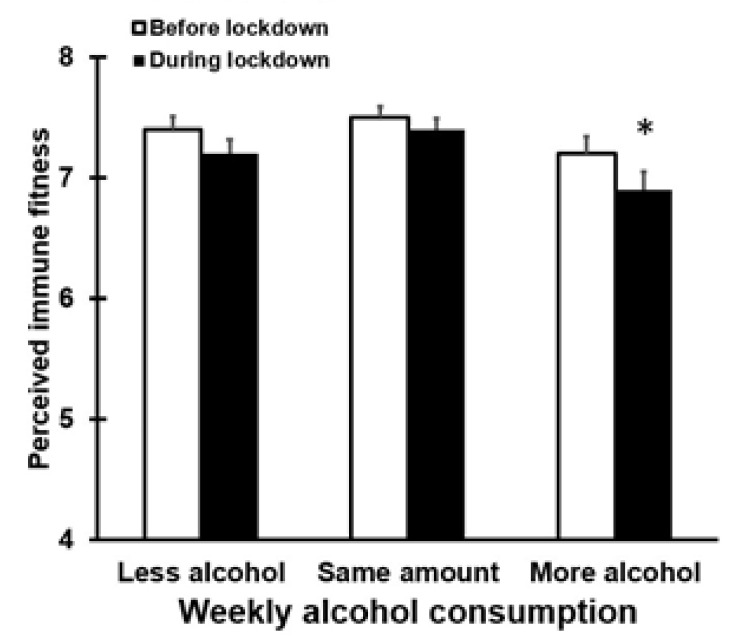
Perceived immune fitness before and during lockdown. Means and standard errors are shown for perceived immune fitness. Nonparametric comparisons were conducted with the independent samples Kruskal–Wallis test. Paired comparisons (two-tailed) were adjusted for multiple comparisons by applying a Bonferroni’s correction. Differences were considered statistically significant if the adjusted *p*-value was < 0.05. Significant differences between the ‘more alcohol’ group and the ‘same amount of alcohol’ group are indicated by *.

When comparing participants who consumed more, less, or the same weekly amounts of alcohol during lockdown, we observed clear differences in reported immune fitness. Figure 4 shows the ratings of perceived immune fitness for participants who that consumed more, less, or the same weekly amounts of alcohol during lockdown. Statistical analysis revealed that perceived immune fitness was significantly different between the groups (*p* = 0.015). Paired comparisons revealed that those who consumed more alcohol during lockdown had a significantly poorer perceived immune fitness compared to drinkers that did not change their weekly alcohol intake (*p* = 0.012).

### 3.4. Alcohol Consumption and the Presence and Severity of COVID-19 Symptoms

**Figure 5:**
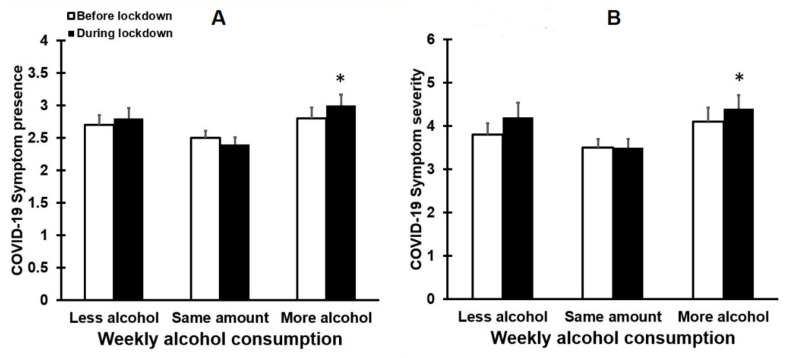
Presence and severity of COVID-19-related symptoms before and during lockdown. Means and standard errors are shown for the presence (**A**) and severity (**B**) of COVID-19-related symptoms. Nonparametric comparisons were conducted with the independent samples Kruskal–Wallis test. Paired comparisons (two-tailed) were adjusted for multiple comparisons by applying a Bonferroni’s correction. Differences were considered statistically significant if the adjusted *p*-value was < 0.05. Significant differences between the ‘more alcohol’ group and the ‘same amount of alcohol’ group are indicated by *.

The presence and severity of COVID-19 symptoms are summarized in Figure 5.

Before lockdown, no significant differences were found between the groups for the presence and severity of COVID-19 symptoms (see Figure 5). During lockdown, however, COVID-19 symptom severity significantly differed between the groups (*p* = 0.013). Paired comparisons revealed that those who consumed more alcohol during lockdown reported significantly more severe COVID-19 symptoms compared to drinkers that did not change their weekly alcohol intake (*p* = 0.013). Moreover, the presence of COVID-19 symptoms differed significantly between the groups (*p* = 0.007). Paired comparisons revealed that those who consumed more alcohol during lockdown reported significantly more COVID-19 symptoms compared to drinkers that did not change their weekly alcohol intake (*p* = 0.007). No significant differences were found between drinkers that reduced their alcohol intake during lockdown and the group that reported the same level of alcohol intake.

### 3.5. Age and Sex as Confounding Factors

As possible confounding factors, age and sex were further evaluated. To this extent, age groups were composed, including 18–25, 26–35, 36–45, 46–55, 56–65, and >65 years old. For drinkers aged 36 and older, no effects on alcohol consumption (frequency and quantity) were found, whereas the 18- to 35-year-old drinkers showed a significant overall decrease in weekly alcohol consumption (mean (SD) number of weekly alcoholic drinks before versus during lockdown were 5.2 (6.5) and 4.6 (6.5), respectively, *p* = 0.030) and a significant overall increase in weekly drinking days (mean (SD) number of drinking days before versus during lockdown were 1.8 (1.3) and 1.9 (1.7), respectively, *p* = 0.044).

**Figure 6:**
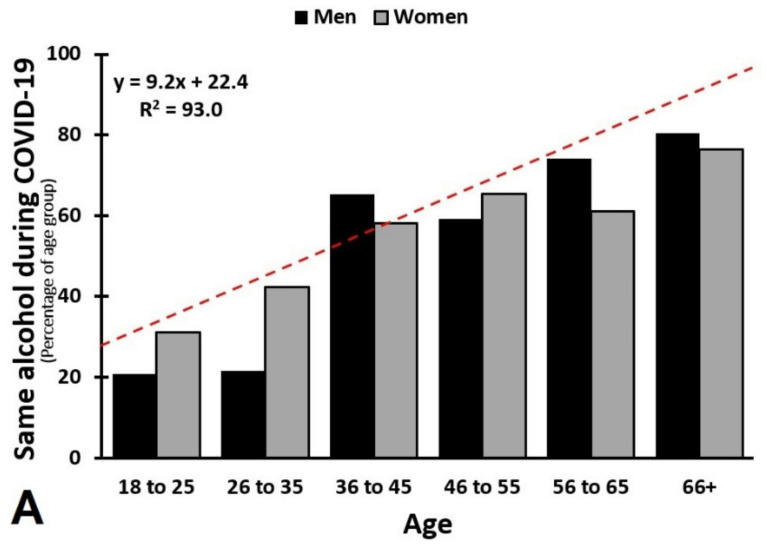

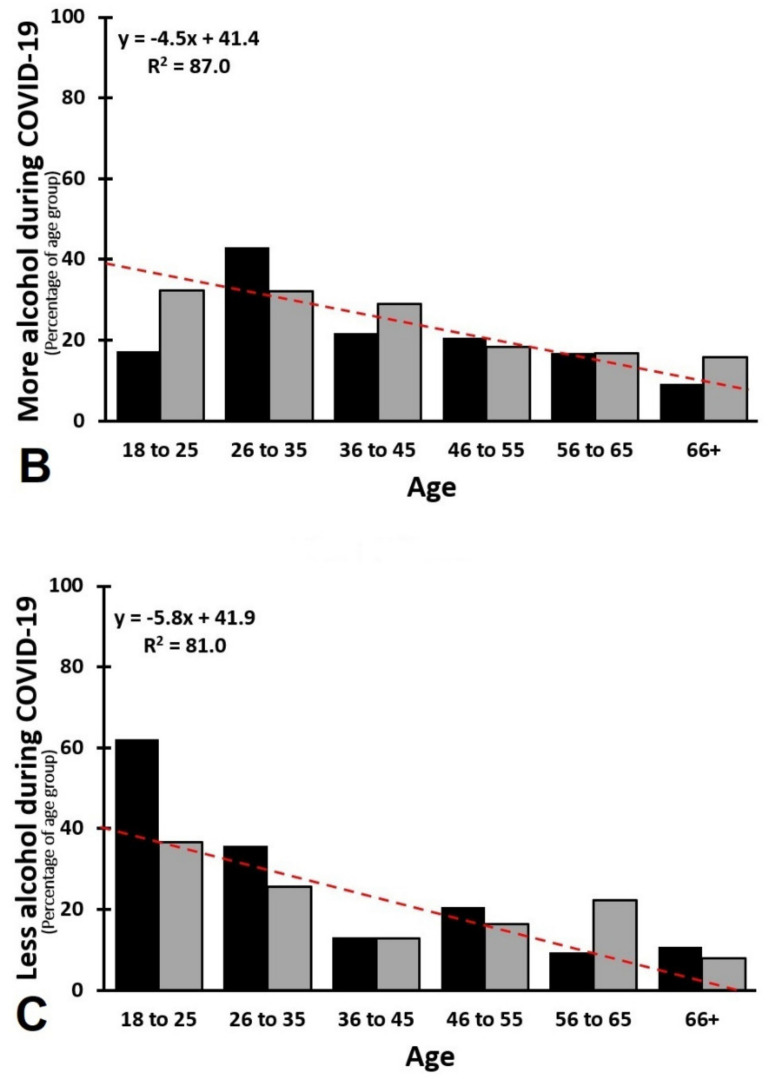
Alcohol consumption according to age and sex. Percentages of participants of each age group are shown for men and women, for participants who drink, compared to before COVID-19, (**A**) the same amount of alcohol, (**B**) more, or (**C**) less alcohol during the COVID-19 lockdown. No significant sex differences were found. Best fitting linear trendlines for age effects were computed and are depicted as red dotted lines.

The effect of age is depicted in Figure 6, showing the percentages of participants that consumed the same, more, or less alcohol during the COVID-19 lockdown. The highly significant trendlines support the age effect. Taken together, the impact of COVID-19 lockdown on weekly alcohol consumption seemed to be limited to 18- to 35-year-old drinkers. Overall, no significant sex difference was found in changes in weekly alcohol consumption (*p* = 0.187). Moreover, after Bonferroni’s correction, across all age groups, no significant sex differences were found in percentages of participants drinking the same, more, or less alcohol.

## 4. Discussion

The current study compared individuals that did not change their weekly alcohol consumption (about half of the sample) with those who either reduced (25.9%) or increased (23.8%) their weekly alcohol use during lockdown. The analysis revealed that both increased and reduced alcohol consumption during lockdown were associated with poorer mood and higher levels of stress. Increased alcohol consumption was associated with significantly reduced perceived immune fitness and a high presence and severity of COVID-19 symptoms. This effect was not significant among individuals with reduced or unaltered alcohol consumption.

Overall, results support and corroborate the association between the COVID-19 pandemic and observed changes in alcohol consumption. The present data show that around half of the 761 social drinkers included in this study either reduced or increased their weekly alcohol intake during lockdown, in line with other COVID-19 studies [26,34,35]. Across the three groups, observations for mood, including anxiety, depression, loneliness, fatigue, hostility, and happiness, as well as stress levels, were significantly poorer during lockdown. Although reduced and increased alcohol consumption during lockdown was associated with poorer mood and higher stress levels, only an increase in alcohol consumption was significantly associated with poorer immune fitness and a higher presence and severity of COVID-19 symptoms.

**Figure 7:**
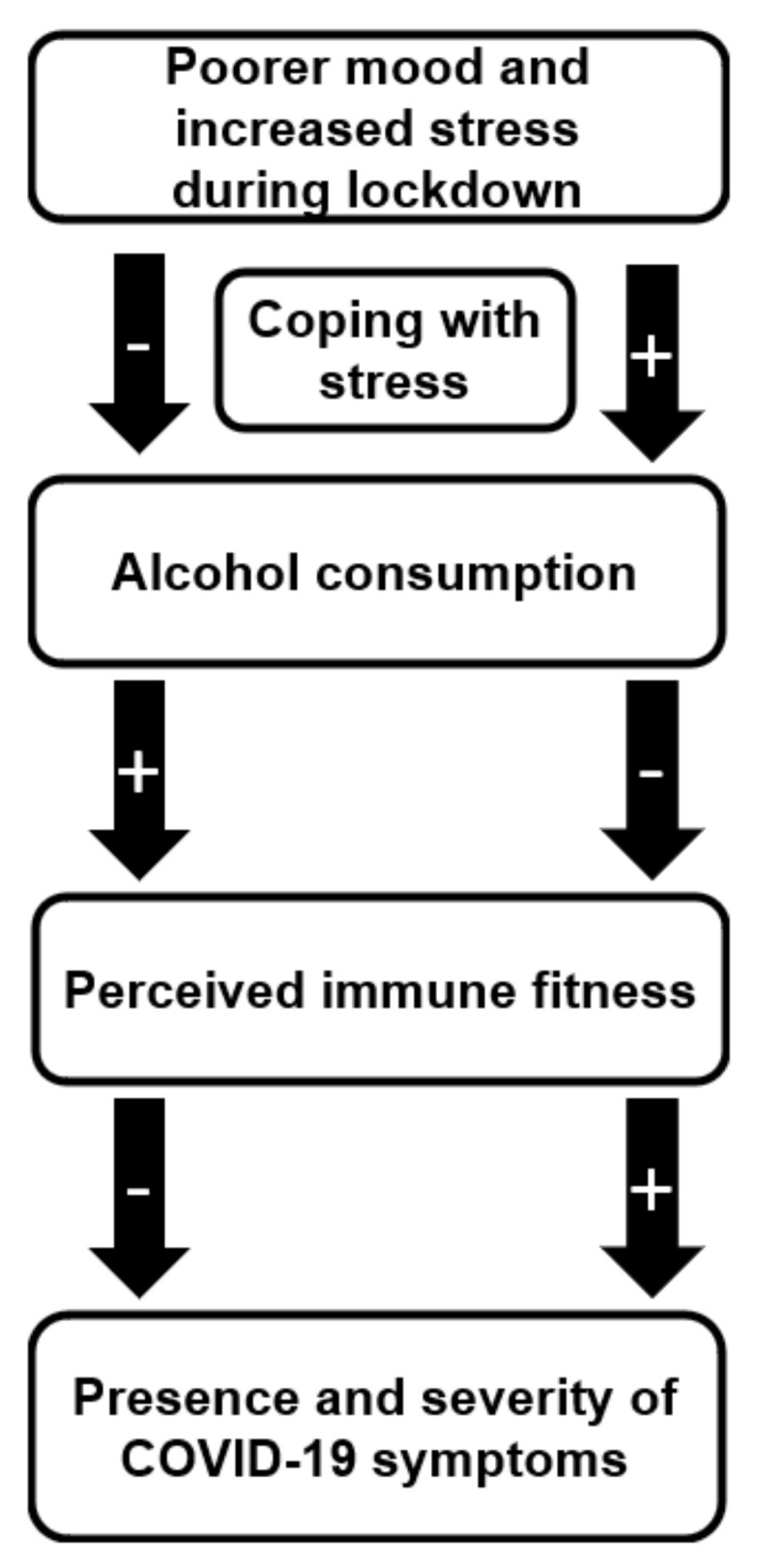
Changes in alcohol consumption in response to alterations in mood and stress during COVID-19 lockdown and its association with perceived immune fitness and the presence and severity of COVID-19-related symptoms. + = positive effect, increase − = negative effect, decrease.

Our findings support the model presented by Merlo et al. [37]. In contrast, a reduction in alcohol consumption was shown to be an adequate coping mechanism for lockdown related mood and stress effects, for maintaining adequate immune fitness, and to reduce their susceptibility of experiencing COVID-19-related symptoms. Our findings, summarized in Figure 7, are in line with previous publications outlined in the introduction, showing that the COVID-19 lockdown and associated mood changes may have differential effects on individuals’ alcohol consumption [15,21,35,36,37].

The association between increased drinking behaviour and poorer mood and depression during the pandemic has also been observed in Australia [26,33], the UK [15], Belgium [22], Greece [27], and the USA [14,23,35]. We observed the same significant associations of a decrease in mood with reduced alcohol intake during lockdown, which have not been previously reported. As there was no significant association between alcohol consumption and perceived immune fitness, or between alcohol consumption and reported COVID-19 related symptoms in this group, it appeared that the reduction in alcohol intake is an adequate coping mechanism to counteract lockdown-related stress and mood changes.

The analysis of age and sex as possible confounders revealed that changes in alcohol consumption were seen in drinkers aged 18 to 35 years old, but not in older drinkers. No significant sex differences were found. Future analyses are underway to evaluate other demographic and personality characteristics of alcohol consumers who cope with COVID-19-related stress by either increasing or decreasing their alcohol consumption.

These findings should be viewed in the context of the study’s limitations. Firstly, the survey data were collected retrospectively by self-report, which could potentially be inaccurate and impacted by recall bias, this being because the time interval between data collection and the (pre-)lockdown assessments was three to six months. The lockdown, however, can be considered an exceptional period, and as such, participants should be able to recall the impact relatively well. It is possible, however, that individuals may have exaggerated the effects of lockdown or even idealized the pre-lockdown period. Prospective studies should therefore confirm our findings. Secondly, immune fitness was assessed by a subjective rating scale [40], and the nature of the study (an anonymous survey) did not allow for conducting of supportive objective assessments of immune functioning (e.g., changes in immune biomarkers). Thirdly, while we assessed the presence and severity of COVID-19 symptoms, at the time the survey was conducted, most Dutch individuals were not actually tested for infection with SARS-CoV-2. Moreover, in the current sample, 77.2% of the sample had not been tested for COVID-19, and of those that were tested, the vast majority (38 of 46 participants) tested negative (i.e., were not infected). Therefore, while poorer immune fitness correlated significantly with increased presence and severity of COVID-19 symptoms, the current data do not allow for any causal inferences. Fourthly, alcohol consumption data were collected as drinks per week and number of drinking days. While this provides important knowledge on overall alcohol consumption, it does not provide information on specific drinking sessions. Moreover, no information was collected concerning the types of alcoholic beverages that were consumed (beer, wine, spirits, or other) and corresponding alcohol levels. Finally, it should be noted that, while the sample comprised participants from both sexes and covered an age range from 18 to 94 years old, it is unclear to what extent the sample of participants is representative for the Dutch drinking population as a whole.

Future studies should evaluate test results. The latter is also important if individuals potentially test positive for COVID-19 whilst being asymptomatic, i.e., not exhibiting any COVID-19 symptoms. Finally, the COVID-19 Symptoms Scale used in this study was designed in the first quarter of 2020. It includes symptoms that at that time were outlined by the Dutch National Institute for Public Health and the Environment (RIVM). However, (from the first quarter of 2021) new knowledge shows that this list of symptoms was incomplete, and thus several symptoms that are currently recognized as core symptoms were not included and evaluated (e.g., loss of taste and smell). Notwithstanding this, significant correlations were found between poorer perceived immune fitness and increased reports of the presence and severity of COVID-19 symptoms. However, future studies should update the COVID-19 symptom listing.

## 5. Conclusions

The aim of this study was to investigate individuals’ mood before and during lockdown who reported consuming more, less, or the same amount of alcohol, and to examine how this impacted perceived immune fitness and the presence and severity of COVID-19-related symptoms. Compared to individuals that did not alter their drinking behaviour, both increased and reduced alcohol consumption during lockdown was associated with poorer mood and higher stress levels. Increased alcohol consumption was associated with significantly reduced perceived immune fitness and a high presence and severity of COVID-19 symptoms. This effect was not significant among individuals with reduced or unaltered alcohol consumption.

## Figures and Tables

**Table 1:** Demographics and study outcomes.

	Overall	Men	Women	*p*-Value
N (%)	761 (100%)	292 (38.4%)	469 (61.6%)	-
Age (year)	42.3 (19.0)	48.0 (19.2)	38.7 (18.0) *	<0.0001 *
Height (m)	1.74 (0.09)	1.81 (0.08)	1.70 (0.07) *	<0.0001 *
Weight (kg)	77.9 (16.8)	85.5 (14.9)	73.1 (16.2) *	<0.0001 *
BMI (kg/m^2^)	25.6 (5.1)	26.0 (4.4)	25.4 (5.4)	0.081

Mean and standard deviation (SD, between brackets) are shown. Significant differences between men and women (*p* < 0.05) are indicated by *. Abbreviations: BMI = body mass index.

## Data Availability

The data are available upon request from the corresponding author.
